# Thrombotic Disease in Hemophilic Patients: Is This a Paradox in a State of Hypocoagulability?

**DOI:** 10.3390/diagnostics14030286

**Published:** 2024-01-29

**Authors:** Oana Viola Badulescu, Minerva Codruta Badescu, Iris Bararu Bojan, Maria Vladeanu, Nina Filip, Stefan Dobreanu, Razvan Tudor, Bogdan-Mihnea Ciuntu, Adelina Tanevski, Manuela Ciocoiu

**Affiliations:** 1 Department of Pathophysiology, Morpho-Functional Sciences (II), Faculty of Medicine, “Grigore T. Popa” University of Medicine and Pharmacy, 700115 Iasi, Romania; violabadulescu@yahoo.com (O.V.B.); maria_apavaloaie@yahoo.com (M.V.); mciocoiu2003@yahoo.com (M.C.); 2 Department of Internal Medicine, Faculty of Medicine, “Grigore T. Popa” University of Medicine and Pharmacy, 700115 Iasi, Romania; 3 Department of Biochemistry, Morpho-Functional Sciences (II), Faculty of Medicine, “Grigore T. Popa” University of Medicine and Pharmacy, 700115 Iasi, Romania; zamosteanu_nina@yahoo.com; 4 Institute of Cardiovascular Diseases, G.I.M. Georgescu, 700503 Iasi, Romania; 5 Department of Orthopedics and Traumatology, Surgical Science (II), Faculty of Medicine, “Grigore T. Popa” University of Medicine and Pharmacy, 700115 Iasi, Romania; rc_tudor@yahoo.com; 6 Department of General Surgery, “Grigore T. Popa” University of Medicine and Pharmacy, 700115 Iași, Romania; bogdanmciuntu@yahoo.com (B.-M.C.); papancea.adelina@yahoo.com (A.T.)

**Keywords:** hemophilia, thrombosis, clotting factors

## Abstract

Hemophilia patients have a deficiency in or dysfunction of clotting factors, which can lead to a bleeding tendency. However, paradoxically, some hemophilia patients may also be at an increased risk of developing thrombotic events such as deep vein thrombosis or pulmonary embolism. The pathophysiology of thrombosis in hemophilia patients is not fully understood, but it is thought to involve a complex interplay of various factors, including the severity of the hemophilia, the presence of other risk factors such as obesity, smoking, or the use of hormonal therapies, and the presence of certain genetic mutations that increase the risk of thrombosis. In addition, it has been suggested that the use of clotting factor replacement therapy, which is a standard treatment for hemophilia, may also contribute to the development of thrombosis in some cases.

## 1. Introduction

Hemophilia is an X-linked recessive genetic disorder that affects the blood’s ability to clot properly due to a deficiency in or dysfunction of clotting factors, primarily factor VIII (hemophilia A) or factor IX (hemophilia B). This leads to prolonged bleeding and bruising, as well as the formation of soft tissue hematomas and joint bleeding, which can result in chronic pain and disability. Hemophilia is caused by mutations in the F8 or F9 genes that encode these clotting factors [1,2,3,4]. 

Hemophilia has been referred to as “the disease of the kings” due to its prevalence among the royal families of Europe, including the descendants of Queen Victoria of England [5,6,7]. The earliest modern documentation of this disorder was by Dr. John Conrad Otto, an American physician, who described an inheritable bleeding disorder affecting only males born from unaffected mothers in several families [4]. Dr. Otto coined the term “bleeders” to refer to these individuals. The term “hemophilia” was first used by Johann Lukas Schönlein in his dissertation at the University of Zurich, Switzerland. Dr. Nasse provided the first genetic description of hemophilia in Nasse’s Law, which postulated that hemophilia is transmitted exclusively by unaffected females to their sons [1,2,5,8].

Venous thromboembolism is a significant contributor to hospital-acquired morbidity and mortality among the hemophilic patient population [9]. Although venous thromboembolism is an exceedingly rare complication in hemophilia patients, a few cases have been reported in the literature [9,10,11]. The majority of these cases involved a triggering event, such as recent surgery, venous catheterization, or the infusion of clotting factor replacement therapy, or the presence of an additional prothrombotic risk factor. However, there have been sporadic reports of exceptional spontaneous thrombotic events in individuals with hemophilia [9,10,11,12].

The underlying mechanism of thrombosis in hemophilia patients is not completely understood, but it is believed to involve a complex interplay of various factors. While individuals with hemophilia typically have a bleeding tendency due to a deficiency in or dysfunction of clotting factors, paradoxically, some may also have an increased susceptibility to thrombotic events such as deep vein thrombosis or pulmonary embolism [9].

## 2. Genetic Mechanisms of Transmission

In 1961, Mary Lyon put forth a hypothesis that during the early stages of development in female mammals, a stochastic and autonomous process leads to the inactivation of one of the two X chromosomes within each cell. This phenomenon results in a mosaic expression pattern, wherein select cells express the X chromosome inherited maternally, while others express the paternally inherited X chromosome.

The purpose of this X chromosome inactivation is to equalize gene dosage between male individuals, characterized by one X and one Y chromosome, and female individuals, characterized by the possession of two X chromosomes. This regulatory mechanism ensures a congruent overall level of gene expression from the X chromosome in both sexes. This proposal offers a more comprehensive insight into the fundamental mechanisms accountable for X-linked disorders [13,14].

As both the F8 and F9 genes are situated on the X chromosome, they are subject to the unique inheritance pattern of X-linked genes, which predominantly affects males. Males with the disease-causing mutation on a single X chromosome are considered hemizygous and display the complete phenotype of the disorder. In contrast, females possess two alleles for X chromosome genes and can compensate for the defect in one allele with the normal allele. As a result, these heterozygous carriers usually maintain a healthy phenotype [14,15,16].

When an affected male has children, his X chromosome is transmitted to his daughters, and his Y chromosome is passed on to his sons [14]. In the event of the male having offspring with an unaffected female, none of his male offspring will be affected by the disease. However, all of his female offspring will be carriers, which are called obligate carriers. By taking into consideration the X-linked inheritance of the disease and family history, it is possible to determine obligate carriers. If a female carrier has a child with a healthy male, each male offspring has a 50% chance of being affected, while each female offspring has a 50% chance of being a carrier. Consequently, the disease can be inherited from affected males to male grandchildren through the carrier daughter [14,16,17].

Around 30% of hemophilia cases, both hemophilia A and hemophilia B, are classified as “sporadic” because the affected child is the first in the family to have the condition. The absence of a family history can be explained by the possibility of a new genetic mutation in the F8/F9 genes that occurred either in the affected child or in a female ancestor who gave birth to a boy with hemophilia, or in her parents. In some cases, a lack of information or a scarcity of male births in the family can obscure the family history of the condition. Maternal mosaicism, in which a genetic mutation is present in some but not all of a mother’s cells, can also contribute to such cases where no mutation is detected in the mother using standard testing techniques. Therefore, a negative family history is insufficient for ruling out hemophilia when clinical symptoms suggest a tendency toward excessive bleeding [14,18].

## 3. Severity of Bleeding in Hemophilia

The severity of bleeding in hemophilia is determined using the degree of deficiency or absence of clotting factors [2,15,19]. Hemophilia can be classified into three levels of severity: severe, moderate, and mild, as summarized in Table 1.

Severe hemophilia is defined by a clotting factor level less than 1% of normal, while moderate hemophilia is defined by a clotting factor level between 1 and 5% of normal. Individuals with severe hemophilia may experience spontaneous bleeding, such as into muscles and joints, as well as prolonged bleeding after injury or surgery. In contrast, individuals with moderate hemophilia typically experience bleeding after injury or surgery but are less likely to experience spontaneous bleeding [2,15,19].

Mild hemophilia is defined by a clotting factor level between 5 and 40% of normal. Individuals with mild hemophilia may experience bleeding after a major injury or surgery, but they typically do not experience spontaneous bleeding. However, individuals with mild hemophilia may still be at risk for bleeding if they have additional risk factors, such as surgery or trauma [2,15,19].

The severity of bleeding in hemophilia can also vary depending on the location of the bleed. Bleeding into joints and muscles can cause pain, swelling, and loss of mobility, while bleeds into organs can be life-threatening.

## 4. Pathophysiology of Hemophilia

**Figure 1:**
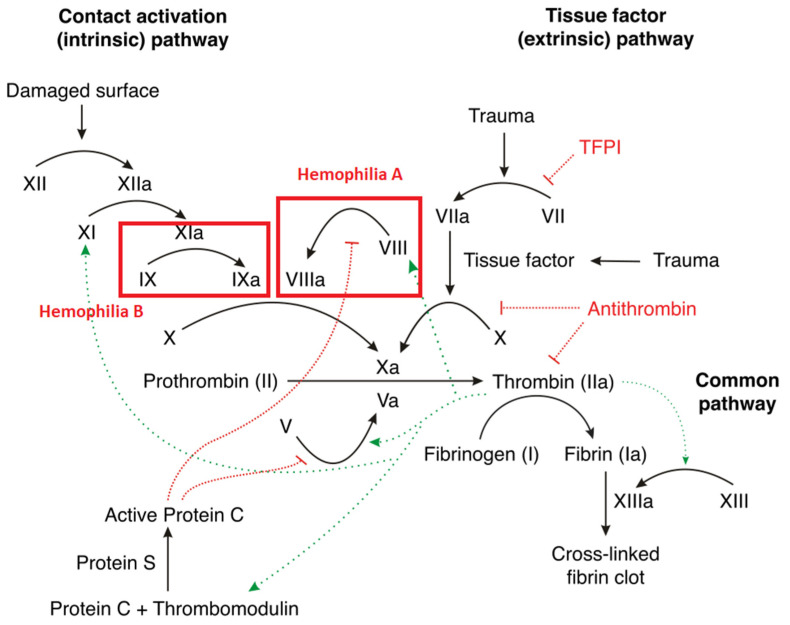
Review of the clotting cascade and the affected clotting factors in hemophilia [17,27]. Hemophilia A is associated with a deficiency in factor VIII. Hemophilia B is associated with factor IX deficiency. TFPI—tissue factor pathway inhibitor.

The bleeding tendency in hemophilia is not solely attributed to the deficiency or absence of coagulation factors VIII or IX [21] and the consequent disruption of the coagulation cascade (Figure 1) [1,17]. Other non-factor-related hemostatic dysfunctions, such as the inability of the extrinsic pathway to compensate for low thrombin generation with the intrinsic defect, local inhibitors such as tissue factor pathway inhibitor in the joints, the up-regulation of the fibrinolytic pathway due to the inadequate activation of thrombin-activable fibrinolysis inhibitor, and the local activators of fibrinolysis such as urokinase-plasminogen activator and tissue-plasminogen activator [22,23,24], also contribute to the pathophysiology of hemophilic bleeding diathesis. Additionally, treatment-related complications, such as FVIII inhibitors, chronic liver disease, human immunodeficiency virus (HIV)-associated thrombocytopenia, NSAID-induced gastritis [25] and thrombocytopathy, and environmentally acquired infectious diseases, including Helicobacter pylori gastritis, intestinal helminthiasis, and urinary schistosomiasis, can exacerbate the bleeding tendency in hemophilia [21,25,26].

Another factor influencing the bleeding tendency in hemophilic patients concerns the primary hemostasis. Protease-activated receptors (PARs) are a subset of membrane receptors responsible for the normal physiological response of platelets to thrombin. Once activated by thrombin cleavage, these receptors trigger intracellular signaling events that transform mobile, non-adhesive platelets into adhesive cells capable of undergoing release reaction and aggregation, culminating in the formation of an immobile hemostatic plug at the site of vascular injury. The recruitment of platelets into this growing plug is dependent on the accumulation of thrombin, which is notably limited in individuals with hemophilia [22,24,26].

## 5. Risk Factors for Thrombosis in Hemophilia

Cardiovascular illnesses are a major contributor to morbidity and mortality in the western world, and hemophilia patients are susceptible to the same cardiovascular risk factors as is the general population [12,28,29,30].

The factors that increase the risk of thrombosis were identified as hypertension (blood pressure greater than 140/90 mm Hg), obesity (Body Mass Index (BMI) greater than 30), hypercholesterolemia (serum LDL greater than 3 mmol/L), smoking, diabetes mellitus, atrial fibrillation (AF), a family history of thrombosis, being HIV-positive, experiencing trauma or major surgery, and receiving recent FVIII/FIX infusion [28,29].

A study conducted in the United States of America by Sharathkumar et al., which included 185 patients, identified a prevalence of cardiovascular disease in hemophilic patients of 19.5%, a value well within the known range of 3–20%, reported in previous studies for the same category of patients [31].

## 6. Potential Mechanisms of Thrombosis

### 6.1. Imbalance of Clotting Factors in Hemophilia

The hemostatic balance in individuals with hemophilia is perturbed due to a deficiency in or malfunction of clotting factors, resulting in an increased propensity for bleeding. Nonetheless, a disequilibrium in clotting factors may also lead to thrombosis in certain instances [32,33]. A reduction in factor VIII may result in a relative increase in the von Willebrand factor, which can in turn promote platelet aggregation and the formation of clots [34].

The Increased release of the von Willebrand factor (vWf) in response to endothelial damage has led to the suggestion that vWf levels may serve as an indicator of endothelial dysfunction. Endothelial dysfunction can alter the ability of cells to participate in coagulation and fibrinolysis, leading to an increased risk of thrombus formation and atherosclerosis [35,36,37]. High levels of vWf have been linked to both thrombogenesis and atherosclerotic vascular disease, which suggests that it may be an indirect indicator of these conditions [34]. The levels of vWf, however, can be affected by various pathological conditions, including the acute phase response [35,36,37].

### 6.2. Platelet Activation

Studies have shown that platelets in hemophilia patients are more susceptible to activation and aggregation compared to platelets in non-hemophilic individuals. This is believed to be due to the compensatory mechanisms that occur in response to the deficient or absent clotting factors. In the absence of adequate clotting factors, platelets may become more reactive in order to compensate for the lack of clot formation [24,38,39,40].

Van Bladel et al. conducted a study which included 21 patients with mild-moderate hemophilia and 13 patients with severe hemophilia. The research revealed that individuals with hemophilia exhibit a higher level of basal-activated platelets in their circulation, respond more robustly to ADP stimulation, and have platelets that are more responsive to inhibition with the prostacyclin analog iloprost compared to healthy individuals. The study also found that patients with hemophilia displayed elevated plasma concentrations of platelet activation markers platelet factor 4 (PF4), chemokine ligand 7 (CXCL7), and RANTES (Regulated upon Activation, Normal T Cell Expressed, and Presumably Secreted), with vWF concentrations being raised in those with severe hemophilia A. Among those with severe hemophilia, platelet activation was found to be correlated with FVIII consumption, indicating that platelet activation is up-regulated in the presence of secondary hemostasis deficiency [24].

The study revealed that patients with hemophilia have elevated baseline levels of platelet activation, indicated by increased platelet P-selectin expression and higher plasma concentrations of the soluble platelet activation markers PF4, CXCL7, and RANTES when compared to healthy controls. This increased platelet activation may compensate for the deficiency in FVIII, as it provides a more negatively charged surface to facilitate the coagulation cascade in hemophiliacs. Additionally, the authors hypothesized that platelet activation would be greater in more severely affected patients. Indeed, the study found that patients with severe hemophilia (residual FVIII < 1%) had higher baseline platelet P-selectin expression compared to those with mild-moderate hemophilia. However, no significant differences were observed in the soluble platelet activation markers PF4, CXCL7, and RANTES between patients with mild-moderate and severe hemophilia [24].

### 6.3. Endothelial Dysfunction

There exists evidence to support the notion that individuals with hemophilia may undergo endothelial dysfunction, which may increase the likelihood of thrombotic events. This can be attributed to the integral role that endothelial cells play in the regulation of coagulation and fibrinolysis. The disruption of these cells may result in a diminished capacity to contribute to these physiological processes, thus predisposing individuals to the development of thrombi and atherosclerosis [12,36,41,42,43].

According to previous research, endothelial dysfunction is recognized as one of the initial indications of atherosclerosis. Measuring the flow-mediated dilation of the brachial artery is a well-recognized approach for assessing endothelial function [12,44].

In a study conducted by Sartory et al. on a population of adult hemophilia patients with mild to moderate-severe disease, it was observed that the majority of the patients had at least one cardiovascular risk factor, and nearly half of them had two or more risk factors. The study also revealed a significant correlation between metabolic parameters associated with insulin resistance and PAI-1, which is involved in the development of atherosclerosis [12].

Endothelial cells are responsible for synthesizing tissue-type plasminogen activator (t-PA), a serine protease that facilitates intravascular fibrinolysis and the removal of fibrin clots. However, active t-PA is quickly inhibited by plasminogen activator inhibitor type 1 (PAI1), which is synthesized by various cell types, including the endothelial and smooth muscle cells found in the vessel wall. Elevated PAI-1 levels are closely associated with insulin resistance indicators and are often observed in patients with metabolic syndrome. Moreover, PAI-1 has been shown to play a role in the development of atherosclerosis and its complications [12,44,45,46].

Glukokalyx shedding and metalloproteinase defects collectively contribute to the intricate pathogenesis of thrombotic events in hemophilia patients, elucidating a multifactorial paradigm [47,48,49]. Glukokalyx shedding, characterized by the disruption of the protective glycocalyx layer lining the endothelial cells, plays a central role in rendering hemophilic individuals susceptible to thrombosis [47,48]. The compromised glycocalyx exposes tissue factor, instigating the coagulation cascade and fostering an environment conducive to abnormal thrombus formation. This process is intricately linked to metalloproteinase defects, which further amplify the vulnerability of hemophilia patients to thrombotic complications [47,48,49,50]. Metalloproteinases, crucial for extracellular matrix remodeling, are integral in maintaining vascular integrity [49,50]. Deficiencies or dysregulation in these proteolytic enzymes contribute to endothelial dysfunction, disrupting the finely tuned balance between pro- and anticoagulant mechanisms, thereby fostering a prothrombotic milieu [51,52,53].

The interplay between glukokalyx shedding and metalloproteinase defects underscores the complexity of thrombotic risk in hemophilia patients. The disrupted glycocalyx and compromised extracellular matrix dynamics collectively create a milieu conducive to aberrant coagulation, emphasizing the need for a comprehensive understanding of these intricate processes [47,48,52,53].

### 6.4. Genetic Factors

Some genetic mutations have been associated with an increased risk of thrombosis in patients and have been proposed to increase the thrombosis risk in hemophilia patients, as well. For example, mutations in the F5 and F2 genes, which encode for clotting factors V and II, respectively, have been linked to an increased risk of thrombosis in hemophilia patients [54,55].

Despite having the same molecular defect, there is a notable clinical phenotype variation among individuals with hemophilia, indicating the involvement of other disease-modulating mechanisms apart from mutations in the F8/F9 genes. Various studies have indicated that the presence of prothrombotic risk factors, including deficiencies in antithrombin, protein C, and protein S and mutations in FV Leiden (FVL) and prothrombin G20210A, can improve the clinical phenotype of hemophilia [14,54,55].

Thrombophilic defects, such as factor V Leiden or the prothrombin variant G20210A, have been linked to not only a less severe hemorrhagic phenotype in hemophilia carriers but also to rare occurrences of thrombotic events [12,55].

Lee et al. conducted a study on 137 subjects which reported that patients with an FVL mutation had lower factor concentrate consumption (mean: 310 vs. 1185 U/kg/y) and fewer bleeding episodes. Other studies have also suggested that individuals with hemophilia who carry the FVL mutation may experience a reduction in the development of hemophilic arthropathy, lower factor concentrate utilization, a delayed occurrence of the first symptomatic bleeding, and reduced bleeding symptoms compared to those without this mutation [19,35,56,57].

The attenuation of the hemophilia phenotype by FVL appears to be linked to a molecular mechanism that involves the reduction in thrombin downregulation through the activated protein C pathway, resulting in an increase in thrombin generation, which in turn may lead to thrombotic complications [19,54].

Fibrinolysis is a crucial physiological process that counterbalances coagulation by degrading fibrin, the main component of blood clots. Several genetic factors can influence fibrinolysis, leading to either hyper- or hypofunctional states [58].

In the context of hypofunction in fibrinolysis, genetic variations may affect key components of the fibrinolytic pathway, such as plasminogen activators and inhibitors. Plasminogen activators, like tissue-type plasminogen activator (tPA), are responsible for converting plasminogen into plasmin, an enzyme that degrades fibrin. Genetic alterations that diminish the expression or activity of these activators can result in impaired fibrinolysis [58,59,60]. Moreover, genetic hypofunction may involve increased levels or enhanced activity of plasminogen activator inhibitors, which impede the conversion of plasminogen to plasmin, thereby inhibiting fibrinolysis. These genetic factors can contribute to a prothrombotic state, where blood clots persist, increasing the risk of thrombotic events [59,60].

Factor XIII plays a crucial role in stabilizing blood clots by cross-linking fibrin strands, contributing to the formation of a durable clot. Likewise, fibrinogen is a key protein involved in blood clot formation, converting into fibrin during the coagulation process [61,62]. In the presence of genetic defects that enhance the hyperfunction of factor XIII or fibrinogen, there is an increased propensity for excessive clot stabilization and resistance to fibrinolysis—the process of clot dissolution. This hypercoagulable state can lead to the formation of more resilient and persistent blood clots, elevating the risk of thrombosis [61,62,63,64].

For hemophilia patients, who typically experience bleeding tendencies due to deficiencies in clotting factors, the coexistence of genetic defects causing hyperfunction in FXIII or fibrinogen introduces a paradoxical challenge. While the primary concern remains bleeding, the hyperactive clotting factors may contribute to a higher likelihood of thrombotic events. Management strategies for hemophilia patients with such genetic defects require a delicate balance, addressing bleeding risks while cautiously considering the potential for thrombosis. This underscores the need for personalized and vigilant therapeutic approaches to mitigate both bleeding and thrombotic complications in these individuals [62,63,64,65].

### 6.5. Viral Infections

A significant portion of individuals with hemophilia who have contracted HIV may also experience venous thromboses, according to various reports in the recent literature [9]. Additionally, a case study has been documented of a recurring deep vein thrombosis (DVT) in an HIV-positive individual with hemophilia [66]. Patients with HIV infection have been found to exhibit various abnormalities in hemostasis that can induce a hypercoagulable state, such as deficiencies in protein C and protein S, antiphospholipid antibodies, lupus anticoagulant, and increased levels of D-dimers and von Willebrand factor. The risk of DVT in these patients may also be linked to a direct effect of the virus on the vascular wall, as high levels of markers for endothelial cell damage, such as soluble thrombomodulin and von Willebrand factor, have been reported. Furthermore, HIV-associated thrombotic thrombocytopenic purpura, potential prothrombotic effects of some protease inhibitors used in antiretroviral therapy, and the role of proinflammatory cytokines that up-regulate tissue factor expression in monocytes and endothelial cells have all been suggested as possible causes of thrombosis in HIV-infected patients [9,58].

In both immunocompetent and immunosuppressed patients, several cases of DVT linked to cytomegalovirus (CMV) infection have been reported. Therefore, a possible causal relationship between CMV infection and venous thrombosis has been suggested. CMV has a direct toxic and inflammatory impact on endothelial cells and can cause thrombotic microangiopathy. Moreover, CMV triggers the expression of tissue factor in endothelial cells and enhances platelet and leukocyte adhesion by altering the endothelium, leading to the formation of thrombi. A vast majority of individuals with hemophilia carry hepatitis C virus markers. Violi et al. observed a higher plasma level of F1 + 2 in patients with liver cirrhosis during HCV infection than during HBV infection. This finding indicates an increased prothrombin activation in HCV-infected patients. Sartori et al. reported a case of portal vein thrombosis in an individual with severe hemophilia A and post-hepatitis cirrhosis [9,66,67,68,69].

### 6.6. Sepsis-Induced Coagulopathy

Sepsis, marked by a systemic inflammatory response to infection, elicits a dysregulated activation of the coagulation system, culminating in disseminated intravascular coagulation (DIC). In the context of hemophilia, a disorder characterized by a deficiency in or dysfunction of clotting factors, the manifestation of sepsis-induced coagulopathies presents a unique challenge as it intersects with the inherent bleeding diathesis [70,71].

The proinflammatory mediators released during sepsis contribute to the aberrant activation of clotting factors, fostering widespread microvascular thrombosis and organ dysfunction. The conventional hemorrhagic predisposition of hemophilia encounters a paradoxical augmentation of thrombotic risk in the milieu of sepsis-induced coagulopathies. The delicate equipoise between hemorrhage and thrombosis is further perturbed, necessitating judicious clinical management [70,71].

### 6.7. Therapy-Related Thrombotic Complications

Hemophilic patients who receive clotting factor replacement therapy are at an increased risk of developing thrombosis. This is due to the use of high doses of clotting factor concentrates, especially in patients with severe hemophilia, which can lead to an imbalance in the coagulation system, resulting in a procoagulant state. The risk is further exacerbated in patients who undergo frequent infusions or prolonged treatment. Additionally, the type of clotting factor concentrate used can also impact the risk of thrombosis, with some factors being more thrombogenic than others. It is therefore essential to closely monitor hemophilic patients receiving clotting factor replacement therapy to mitigate the risk of thrombosis and adjust the treatment plan accordingly [69,72,73].

Most of the existing evidence on thrombotic complications in hemophilic patients receiving clotting factor replacement therapy is limited to small observational studies or single case reports. One of the few systematic reviews on this topic, conducted over a decade ago by Coppola et al., which included 5579 patients, of which there were 4420 with hemophilia A, 748 with hemophilia B, and 361 with von Willebrand disease, reported a very low incidence of thrombosis in hemophilic patients receiving factor replacement therapy, with the majority of cases being superficial thrombophlebitis. Only a few major venous thromboembolic events were reported, both in VWD patients who had received prolonged factor replacement for surgery and were associated with abnormally high FVIII levels. In contrast, seven VWD patients developed venous thrombosis and/or pulmonary embolism, mostly following prolonged replacement due to severe bleeding or major surgery and in the presence of coexisting risk factors such as elderly age, estrogen intake, and obesity [69]. The thrombotic complications are summarized in Table 2.

The unexpected occurrence of both arterial and venous thrombosis in patients with congenital bleeding disorders has also been documented in clinical trials of non-factor therapies. This highlights the delicate balance between hemostasis and thrombosis and the potential risk of thrombosis in hemophilic individuals who receive both non-factor therapy and standard factor for acute bleeds [74,75]. During an early phase trial of emicizumab in hemophilia A patients, a total of seven thrombotic events were reported, including three cases of thrombotic microangiopathy in patients who received APCCs >100 U/kg within 24 h for acute breakthrough bleeds. Furthermore, there were three instances of venous thrombosis and one of arterial thrombosis, with ongoing investigation into the types and sites of the remaining thromboses [76].

The AFFINE trial is a phase III study designed to assess the clinical efficacy and safety of a single iv infusion of giroctocogene fitelparvovec in adult male participants with moderately severe or severe hemophilia A, with an anticipated enrollment of around 50 participants. Over 50% of the participants in the trial have already received their dose of giroctocogene fitelparvovec, a genetic treatment that utilizes recombinant adeno-associated virus serotype 6 vector (AAV2/6) encoding the complementary deoxyribonucleic acid for B domain deleted human FVIII. However, the trial was put on hold between November 2021 and September 2022 by the Food and Drug Administration in order to implement a protocol amendment due to observations of factor VIII levels exceeding 150% in some participants during the trial [76,77].

As of now, no participant who has received this gene therapy has experienced a thrombotic event. Some participants are being administered oral anticoagulants to minimize the risk of thrombosis. The protocol amendment is intended to provide guidelines for the clinical management of elevated factor VIII levels in order to ensure the safety of participants in the trial [78,79,80].

## 7. Conclusions

Thrombotic disease is a rare but serious complication that can arise in patients with hemophilia, a genetic disorder that impairs the blood’s ability to clot properly, thereby increasing the risk of bleeding. In some instances, hemophilia patients may develop thrombotic conditions, characterized by the accumulation of blood clots in the venous or arterial vessels, posing a significant risk for severe complications such as myocardial infarction, cerebrovascular accident, and pulmonary embolism. The etiology of thrombotic disease in hemophilia patients is considered multifactorial, with several factors believed to contribute to its onset, including hemophilia severity, coexisting risk factors such as obesity, smoking, or hormonal treatment, and specific genetic mutations that can heighten the risk of thrombosis. 

There are several proposed mechanisms for thrombosis in hemophilia, although the exact underlying processes are not fully understood. One theory suggests that hemophilia itself may contribute to a prothrombotic state by causing alterations in the endothelium, the cells lining the blood vessels. 

Other proposed mechanisms include the potential impact of treatment with clotting factor replacement therapy, which can increase levels of procoagulant factors, and the presence of coexisting risk factors such as obesity, smoking, or hormonal treatment.

The pathophysiology of thrombosis in hemophilia patients is a complex and not fully understood process. Further research is needed to elucidate the underlying mechanisms and develop effective prevention and treatment strategies for thrombotic events in this population.

## Figures and Tables

**Table 1:** Summarized classification of the bleeding severity in hemophilia [19,20].

Severity	Clotting Factor Level	Bleeding Episodes
Mild	>5%	Rare, often after surgery or trauma
Moderate	1–5%	Occasional, usually with injury or surgery
Severe	<1%	Spontaneous, frequent, and severe bleeding

**Table 2:** Summary of thrombotic complications in correlation with the administered treatment [69].

Bleeding Disorder	Patients	Type of Product	VTE	Thrombo-Phlebitis	Thrombotic AEs/Patients (%)	Thrombotic AEs/ Total AEs (%)
Hemophilia A	4420	pdFVIII or rFVIII	0	2	2/4420 (0.045)	2/423 (0.47)
Hemophilia B	748	pdFIX or rFIX	0	11	11/748 (1.47)	2/104 (1.92)
von Willebrand disease	361	pdVWF/FVIII or rVWF/FVIII	2	5	7/361 (1.94)	14.0
All	5528	pd and r products	2	18	20/5528 (0.36)	1.91

Note: pd = plasma-derived, rFVIII = recombinant factor VIII, rFIX = recombinant factor IX, VTE = venous thromboembolism.

## Data Availability

Not applicable.
